# A Comparison of Periodontal Health in Elderly Individuals with and Without Metabolic Syndrome: A Retrospective Study

**DOI:** 10.3390/medicina61122200

**Published:** 2025-12-12

**Authors:** Jacqueline LeNoir, Leticia Lenoir, Ahmed Khocht

**Affiliations:** 1 Private Practice, Albany, NY 12203, USA; jlenoir001@gmail.com; 2 School of Dentistry, Loma Linda University, Loma Linda, CA 92350, USA; llenoir@llu.edu

**Keywords:** metabolic syndrome, periodontitis, elderly, radiography, bone resorption

## Abstract

*Background and Objectives*: Metabolic syndrome (MetS) is a cluster of metabolic conditions that increase the risk of heart disease, stroke, and diabetes. This study aimed to test the hypothesis that MetS is associated with an increased risk of periodontitis in elderly individuals. *Materials and Methods*: The Loma Linda University (LLU) institutional review board approved the conduct of this study. Search queries scanned LLU School of Dentistry patient records to identify dentulous individuals ≥ 60 years, with a minimum of 15 teeth and a full mouth series of digital periapical radiographs. Out of 3534 records of individuals ≥ 60 years, random sampling selected 40 MetS cases and 40 healthy controls. Fifteen records were discarded due to inadequate radiographs. The concurrent presence of central obesity, type 2 diabetes, and hypertension was used to determine MetS status. Clinical periodontal measures were collected, and available radiographic records were used for alveolar bone measurements. *Results*: Probing depths ≥ 4 mm and bleeding on probing were significantly higher in the MetS group than the healthy group. Average alveolar bone levels, as well as sites with ≥4 mm bone loss, were also significantly higher in the MetS group than the healthy group. Multivariate analyses adjusting for demographics (age, gender, race), cigarette smoking, and plaque scores confirmed the adverse impact of MetS on periodontal measures. *Conclusions*: Elderly individuals with MetS have an increased risk of periodontal disease.

## 1. Introduction

Periodontitis is a chronic multifactorial inflammatory disease initiated by dysbiotic plaque biofilm in the dentogingival region [1]. It is characterized by the progressive deterioration of tooth supporting structures. Its main features are gingival bleeding, periodontal pocketing, and the loss of periodontal tissue support, which is shown clinically by attachment loss and radiographically by alveolar bone loss. Periodontitis is a significant public health concern due to its high prevalence, the possibility for tooth loss, its detrimental effects on chewing function and appearance, and its potential to lower quality of life. A large percentage of edentulism and masticatory dysfunctions are caused by periodontitis, which also raises the expense of dental care and may be detrimental to overall health.

Subgingival dysbiotic microbial communities of keystone pathogens and pathobionts engage in synergistic virulence, whereby they can both survive the host response and flourish by taking advantage of tissue-induced inflammation; this triggers a self-feeding cycle of escalating dysbiosis and inflammatory bone loss, potentially leading to tooth loss and systemic complications [2]. Recruited neutrophils to the gingival crevice fail to control the dysbiotic microbiota, which can thus invade the connective tissue and interact with other innate immune cell types, such as macrophages and dendritic cells [3]. These cells produce pro-inflammatory mediators [such as TNFa, IL-1b, IL-17] and also regulate the development of adaptive immunity, which also contributes to and exacerbates the inflammatory response [3]. Activated lymphocytes (B and T cells) play a major role in pathological bone resorption through the expression of the receptor activator of nuclear factor κB ligand, which drives the maturation of osteoclast precursors and eventually leads to alveolar bone resorption [2]. Multiple systemic conditions, including obesity, diabetes, and hypertension, increase the risk for periodontitis.

Periodontitis is a quite common disease, affecting approximately 47.2% of adults in the United States aged 30 years and older [4]. The prevalence and severity of periodontitis increase with age. A study that examined periodontitis in US adults aged 65 and older showed that, on average, 66.2% had periodontitis [5]. The reasons for the increased prevalence and severity of periodontitis in the elderly are not well understood.

Metabolic syndrome (MetS) is a cluster of medical conditions that increase the risk of developing chronic diseases such as heart disease, stroke, and type 2 diabetes. It is defined by having three or more of the following factors: abdominal obesity, high blood pressure, high fasting blood sugar, high triglycerides, and low HDL cholesterol [6]. MetS and associated obesity have been linked to periodontal disease [7,8,9]. Jepsen et al. 2020 suggested that there is not only a causal, but a potentially bidirectional relationship between periodontitis and MetS [10]. Multiple recent studies showed increased incidence of MetS and the number of MetS components in individuals with periodontitis [11,12,13].

Both MetS and periodontitis share common risk factors, such as socioeconomic status, lifestyle/reduced health awareness, and aging [10]. Additionally, both diseases are associated with systemic inflammation [10]. Various inflammatory markers are higher in patients with periodontitis and tend to decline after periodontal therapy. Additionally, subgingival bacteria may influence systemic inflammation. Low-grade chronic inflammation is also an important characteristic of MetS that has been recognized as a major contributing factor to its development and related pathophysiological effects, including insulin resistance. MetS-related inflammation may be caused by dietary choices, adipocyte malfunction, adipose tissue derived cytokines, and dysmetabolism. Collectively, the evidence suggests that subgingival microbial dysbiosis and an elevated inflammatory state serve as a shared pathogenic route in both diseases.

The prevalence of MetS, including type 2 diabetes, obesity, hyperlipidemia, and hypertension, has increased from 1988 to 2012 for every socio-demographic group, with the highest burden observed among non-Hispanic white women, non-Hispanic black women, and adults with low socioeconomic status. By 2012, more than one-third of all US adults met the criteria for MetS [14]. This increase, however, was not solely driven by the rising prevalence of obesity among US adults. Rather, MetS prevalence remained relatively constant over time, even among the non-obese [14].

Furthermore, the prevalence of MetS, like periodontitis, also increases with age. As the US population ages, the rates of both periodontitis and metabolic disease are likely to continue to increase. The number of elderly people, or people aged 65 and older, in the United States on 1 July 2015 accounted for 14.9 percent of the total population in the United States. The projected population of elderly individuals in 2060 is expected to comprise nearly one in four U.S. residents [15].

The impact of MetS on periodontal health in the elderly population has not been thoroughly investigated. To address this knowledge gap, the present investigation evaluates the relationships between MetS and periodontitis in the elderly. The constellation of morbidities associated with MetS may offer valuable insights into the collective impact of diseases such as obesity, hypertension, and diabetes on periodontal health. We hypothesized that the high prevalence of MetS-related perturbations in systemic health among the elderly may increase their risk of periodontal disease. To test our hypothesis, we compared periodontal health measures between elderly individuals with and without MetS.

## 2. Materials and Methods

The study protocol was reviewed and approved by the LLU Institutional Review Board. Search queries scanned the LLU School of Dentistry patient health records for a 4-year period (2016–2020) to identify all dentulous individuals with a minimum of 15 teeth and a full mouth series of digital periapical radiographs. Records of dentate individuals who satisfied the above stated study inclusion criteria were identified. The following data were collected: demographics (age, gender, and race), anthropometric measurements, existing MetS-related medical conditions (diabetes type 2, hypertension, obesity), cigarette smoking status, and periodontal clinical data (percent plaque score, percent bleeding on probing (BOP%), percent of sites with probing depth (PD) ≥ 4 mm, and number of missing teeth). BMI was calculated from anthropometric measurements. Central obesity was determined with the photographic figure rating scale applied to existing photographs [16]. The concurrent presence of central obesity, type 2 diabetes, and hypertension was used to determine MetS status.

**Figure 1:**
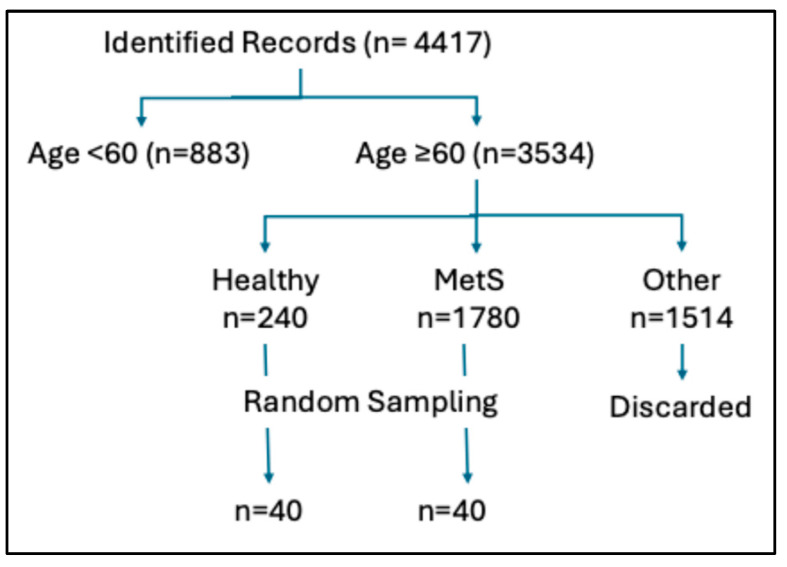
Flow chart showing processing of selected records.

The identified records were divided into two groups according to age category: <60 years and ≥60 years (the study’s focus); see Figure 1. Next, the ≥60 year records were further subdivided into three subgroups: healthy (no existing medical conditions), MetS (concurrent presence of central obesity, type 2 diabetes, and hypertension), and other medical conditions not meeting criteria for MetS. Records of individuals ≥ 60 years with medical conditions not meeting criteria for MetS were discarded. Random sampling (JMP Statistical Package, version 18.2) was used to select 40 records from the ≥60 year MetS subgroup and 40 records from the ≥60 year healthy subgroup for periodontal measure comparisons. The most recent full mouth series of digital intraoral radiographs belonging to the randomly selected records were used for radiographic alveolar bone assessments.

MiPACS Dental Enterprise Viewer software (version 4.3) was used for radiographic alveolar bone assessments. The alveolar bone level (ABL) on interproximal sites of all teeth present was measured from periapical radiographs. Individual radiographs were viewed at full-screen magnification. The distance from the cementoenamel junction (CEJ) to the most coronal point where the periodontal ligament space remained at a normal width was measured in millimeters. The calibrated digital ruler tool was used to record all radiographic measurements. The examiners independently assessed all radiographic records twice, and the average of both assessments was used for data analysis. Images with poor diagnostic quality were excluded. Examiners were blinded to MetS status at the time of radiographic assessments.

Examiner calibration: The study examiners discussed and reviewed the process of alveolar bone level assessments over multiple sessions (1 h each). They defined alveolar bone crestal level, cementoenamel junction (CEJ), and the most coronal point where the periodontal ligament space remained at normal width. They practiced together and compared measurements on sample images. Prior to actual data collection, a series of full mouth radiographs (14 images) were measured twice separately by all examiners. Measurement system analyses showed a high examiner repeatability and reproducibility. Intraclass correlation was 0.97. Gage Repeatability and Reproducibility results showed that the percent of total variance was 6.88%, with repeatability at 6.81% and reproducibility at 0.07%.

Statistical analysis: The power analysis was primarily based on the assumption that MetS would adversely affect alveolar bone levels. It indicated a need for 29 radiographic records per group to be able to assess a difference of 0.5 SD of bone level difference between the two groups at 80% power. We oversampled by approximately 30% in anticipation that some radiographic records may not be usable. Age was normally distributed. The distributions of BMI, waist circumference, and all periodontal measures were positively skewed. Significant differences among the groups for age were determined using Student’s *t*-test, for non-parametric continuous measures using the Wilcoxon rank-sum test, and for categorical variables using the likelihood ratio chi-squared test. Regression analysis was also used for the comparison of periodontal measures between the two groups while adjusting for demographics (age, gender, and race), cigarette smoking, and plaque scores. Rank transformation was used in linear regression models [17].

## 3. Results

**Figure 2:**
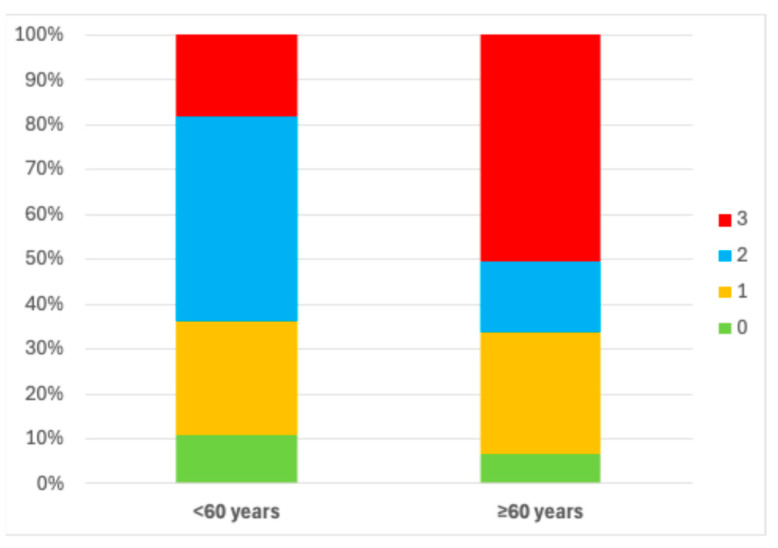
Stacked bar chart showing percentage of individuals with MetS-related medical conditions by age group: 0 (no exiting MetS conditions), 1 (single MetS condition), 2 (two MetS conditions), and 3 (three or more MetS conditions). Likelihood ratio test showed that percentage of individuals with multiple MetS-related medical conditions was significantly higher in ≥60 years than <60 years, X^2^ (3) = 450.37, *p* < 0.00001.

A total of 4417 records of dentulous individuals were obtained; 883 (20%) were <60 years old and 3534 (80%) were ≥60 years old. Figure 2 shows that, in both age groups, most individuals had MetS-related medical conditions. However, individuals aged ≥60 years showed a greater prevalence of such conditions than individuals aged <60 years. Thus, our data show increased rates of MetS in the elderly.

Table 1 summarizes the characteristics of the randomly selected records for periodontal measures comparisons. Fifteen records (eight MetS and seven healthy) were discarded due to inadequate radiographs. The healthy group was slightly younger than the MetS group. Both groups were statistically comparable on gender, race, and cigarette smoking status. Very few subjects in both groups were current smokers. As would be expected, BMI and waist circumference were significantly higher in the MetS group than the healthy group. All MetS individuals had a history of type 2 diabetes and hypertension.

**Figure 3:**
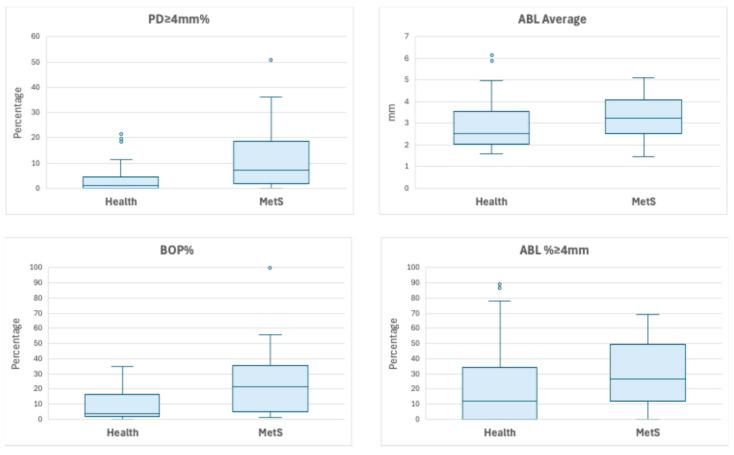
Box plots of PD ≥ 4 mm%, BOP%, ABL average, and ABL % ≥ 4 mm by group (healthy vs. MetS). The bottom and top sides of each box represent the lower and upper quartiles, respectively. The line inside the box represents the median. The bottom and top whiskers represent the minimum and maximum values, respectively. Outliers (values beyond the upper and lower bounds) are marked with dots. Wilcoxon two-sample test showed significantly higher levels in MetS group for PD ≥ 4 mm% (S = 1300.5, *p* < 0.001), BOP% (S = 697, *p* < 0.0005), ABL average (S = 1215, *p* < 0.03), and ABL % ≥ 4 mm (S = 1226, *p* < 0.02).

Table 2 summarizes periodontal measures. PI and the number of missing teeth were statistically comparable between the two groups. PD ≥ 4 mm and BOP were significantly higher in the MetS group than the healthy group. ABL average measures and ABL% ≥ 4 mm were significantly higher in the MetS group than the healthy group. Figure 3 illustrates the differences between the MetS group and the healthy group for PD, BOP, ABL average, and ABL% ≥ 4 mm.

Multivariate analyses adjusting for demographics (age, gender, race), cigarette smoking, and plaque scores confirmed the adverse impact of MetS on periodontal measures (PD, BOP, ABL average, and ABL % ≥ 4 mm) (Table 3).

## 4. Discussion

This study assessed the impact of MetS on periodontal health in the elderly. We compared clinical and radiographic periodontal health measures between elderly individuals with and without MetS. All assessed periodontal measures were significantly worse in MetS individuals than medically healthy individuals. The findings support our hypothesis that MetS is an important factor for the increased susceptibility to periodontitis among elderly individuals.

There are multiple definitions of MetS, including those of the National Cholesterol Education Program’s Adult Treatment Panel III (ATP III) and the International Diabetes Federation (IDF) [18]. The primary difference is that the IDF definition of MetS often results in a higher prevalence than the ATP III definition, mainly because the IDF definition uses lower, ethnicity-specific waist circumference cutoffs as a mandatory component. The IDF definition requires central obesity for diagnosis, while ATP III includes it as one of five components that must be met. This difference in criteria can lead to the two definitions identifying different individuals as having MetS. For the purposes of this study, we mainly focused on MetS components with known associations with periodontitis, such as obesity, hypertension, and diabetes.

Our study showed increased rates of metabolic syndrome in the elderly. This is likely due to age-related physiological changes such as decreased metabolic rate and hormonal shifts, cumulative exposure to lifestyle factors, and the higher prevalence of central obesity. Additionally, chronic low-grade inflammation and shifts in body composition, such as the loss of muscle mass and gain of visceral fat, are major contributors that increase susceptibility to conditions like insulin resistance and type 2 diabetes.

As people age, their metabolism slows down, which can lead to weight gain, especially around the abdomen [19]. Hormonal alterations in elderly women can lead to hormonal changes that increase the risk of a larger waistline, high blood sugar, and low “good” HDL cholesterol. Aging is also associated with a decline in muscle strength and mass, which impacts metabolic function and insulin resistance. A lifelong pattern of physical inactivity or a decrease in activity in later life is a significant risk factor for metabolic syndrome. The cumulative lifetime exposure to various environmental and lifestyle factors over a lifetime can also increase the risk of developing metabolic syndrome in old age.

The immune system in older adults is more prone to chronic low-grade inflammation, which is linked to metabolic dysfunction and an increased susceptibility to diseases like type 2 diabetes and cardiovascular disease [20]. Increased oxidative stress, an important driver of inflammation, also plays a role in the development of metabolic syndrome. A higher prevalence of visceral obesity is a key factor in metabolic syndrome among the elderly. Waist circumference can be a better indicator than BMI in this age group due to muscle loss and height reduction, which can make BMI measurements less accurate.

The association of metabolic syndrome with periodontal disease was previously investigated in several studies. Nesbitt et al. (2010) examined the association of periodontitis with MetS, with and without a consideration of systemic inflammatory status [21]. Participants with radiographic evidence of moderate to advanced alveolar bone loss were significantly more likely to have MetS than those with minimal or no bone loss. No significant differences in systemic inflammation were found between periodontal groups. The authors suggested that the association of alveolar bone loss with MetS is consistent with the hypothesis that destructive periodontal disease may contribute to the development of MetS and elevations in systemic inflammation.

Kaye et al. (2016), in a prospective study, investigated whether metabolic syndrome predicts tooth loss and the worsening of periodontal disease in a cohort of 760 men who were followed up to 33 years [22]. Metabolic syndrome increased the hazards of tooth loss, pocket depth ≥ 5 mm, clinical attachment loss ≥ 5 mm, alveolar bone loss ≥ 40%, and tooth mobility ≥ 0.5 mm. The number of positive metabolic syndrome conditions was also associated with each of these outcomes. Pham (2018) investigated the association between periodontal disease severity and metabolic syndrome (MetS) in a group of Vietnamese patients [23]. Severe and extensive periodontal disease was found in MetS participants and increased with number of MetS components. Participants with higher periodontal parameters had a higher risk of MetS.

Tegelberg et al. (2019) investigated whether metabolic syndrome (MetS) is associated with deepened periodontal pockets and alveolar bone loss [24]. The relative risks for PD ≥ 4 mm and bone loss ≥ 5 mm were higher in individuals with an exposure to MetS ≥ 15 years than in those whose exposure was <15 years. Consistently stronger associations were found in never smokers. They concluded that a long-term exposure to MetS was associated independently with measures of periodontal disease progression. Hlushchenko et al. (2020) surveyed 190 people with metabolic syndrome, with an age range from 25 to 55 years [25]. Periodontal disease was detected in 155 of 190 patients with metabolic syndrome (approximately 81.58%). Generalized moderate to advanced periodontitis prevailed among patients with metabolic syndrome.

More recently, Li et al. (2025) explored the association between periodontitis and MetS prevalence and evaluated its impact on the prognosis of all-cause and cardiovascular mortality in MetS [12]. A total of 9270 individuals were included in the analysis. Individuals with periodontitis had a higher prevalence of MetS compared to those without periodontitis. Periodontitis was associated with an increased risk of all-cause mortality and cardiovascular mortality among individuals with MetS. Our findings agree with previously reported research and expand this knowledge into the elderly population.

Obesity is a chronic, low-grade inflammatory disorder. White adipose tissue plays an important role in regulating systemic energy homeostasis. In obesity, white adipose tissue may turn dysfunctional and not expand properly to store the energy excess. Enlarged visceral adipose tissue is infiltrated with pro-inflammatory immune cells (e.g., M1 macrophages and cytotoxic T cells) and secretes excessive amounts of pro-inflammatory adipokines (e.g., adiponectin, leptin, visfatin, resistin) and cytokines (e.g., TNF-α, IL-1β, and IL-6) [26]. These adverse inflammatory signals induce a state of chronic systemic inflammation and may contribute to the development of insulin resistance, metabolic dysregulation, immune dysfunction, and other obesity-associated disorders [27].

Periodontitis is prevalent among obese individuals [28]. Also, periodontal treatment outcomes in obese individuals are poor [29]. Inflammatory cytokines and adipokines released from enlarged dysfunctional adipose tissue find their way to gingival tissues, which may partly explain the association between obesity and periodontitis [30]. Of particular interest is visfatin, which has been associated with the subgingival colonization of Porphyromonas gingivalis [31]. We and others have shown that obesity promotes subgingival dysbiosis [32]. Obesity-related oxidative stress contributes to gingival tissue inflammation and probably encourages subgingival microbial dysbiosis. Furthermore, obesity drives immune dysfunction and infection development.

Type 2 diabetes contributes to periodontitis primarily through higher blood glucose levels, which leads to vascular dysfunction, induces oxidative stress, weakens the immune system and promotes subgingival bacterial dysbiosis [33]. The combination of these factors makes it harder for the body to fight periodontal infections, while also increasing inflammation and accelerating periodontal tissue damage. Vascular dysfunction associated with type 2 diabetes slows the flow of nutrients to periodontal tissues and the removal of waste products [34]. This weakens periodontal tissue resistance and increases vulnerability to infection.

Type 2 diabetes may also adversely influence inflammatory cytokine expression in gingival tissues, which can further periodontal disease progression and the development of complications [35]. Advanced Glycation End products (AGEs) and their receptor (RAGE) are central to this link, creating a cycle where AGEs trigger inflammation, which then damages cells, contributing to hyperglycemia and other complications [36]. The high levels of inflammatory markers in periodontal tissues induces the production of degrading enzymes that destroy gingival collagens and promote osteoclast activation and alveolar bone loss. Like diabetes, periodontitis also involves inflammation. This can create a vicious cycle where inflammation from one condition worsens the other. The chronic inflammation and infection from periodontitis can, in turn, make it more difficult to control blood sugar levels and exacerbate the diabetic state.

Hypertension (HTN), also known as high blood pressure, is a condition in which the blood pressure in the arteries is consistently elevated above normal levels. HTN is diagnosed when the systolic pressure is consistently ≥140 mm Hg or when the diastolic blood pressure is consistently ≥90 mm Hg [37]. Lifestyle factors like smoking, obesity, lack of physical activity, and high salt intake contribute to HTN development. HTN is linked to disrupted collagen metabolism [38] and heightened systemic inflammation and oxidative stress [39] and serves as a risk factor for various disorders, including heart failure and stroke.

Multiple epidemiologic studies showed that individuals with HTN have an increased risk for periodontitis [40,41]. Animal studies indicated that HTN diminishes the quality of tooth-supporting bone and correlates with ongoing alveolar bone loss, even after the removal of the stimulus for periodontal disease induction [42]. The exact mechanisms linking HTN with periodontitis are not known. HTN-related oxidative stress and inflammation may alter collagen metabolism and weaken gingival tissue resistance. We have shown that individuals with elevated blood pressure have increased levels of the inflammatory mediators CRP and TNFα in the gingival fluid collected from non-diseased sites, which may exacerbate the gingival inflammatory response to periodontopathic bacteria, leading to periodontal tissue damage [43].

The strengths of this study include a diverse cohort of elderly individuals ranging in age from early 60s to late 90s, with both sexes, multiple ethnicities, and all periodontal disease stages represented. The study limitations include a reliance on periodontal charting records taken from multiple uncalibrated providers. The available radiographs were non-standardized as well and taken under normal everyday clinical practice conditions. Central obesity was determined using a photographic figure rating scale and self-reported anthropometric data. Important confounders such as medication use, dental visit frequency, oral hygiene support, alcohol consumption, lipid profiles, socioeconomic status, and cumulative smoking exposure were not included. Furthermore, the study population consists of dental clinic attendees, who may have higher rates of both MetS and periodontal disease than the general elderly population. The study sample included only those records with available full mouth intraoral radiographs. Despite these limitations, relevant associations were noted between periodontal health measures and MetS which further our understanding of how a conglomerate of chronic medical conditions may impact periodontal health.

Future studies correlating measures of systemic inflammation and gingival fluid biomarkers with measures of periodontal health could provide a deeper understanding of the biological mechanisms involved with periodontitis in the elderly population with chronic medical conditions. Longitudinal studies to evaluate the outcome of periodontal therapy in the elderly with chronic medical conditions can help with defining treatment outcome expectations. Such directions can inspire novel therapies and can further advance the field of periodontal research in elderly patients.

Based on the study’s findings, focused periodontal care is essential for elderly individuals to improve oral hygiene, manage periodontal disease, and minimize tooth loss. Practical recommendations to improve access to periodontal care for the elderly include expanding in-home services and telehealth and making home modifications to improve oral hygiene, with a focus on aids to overcome dexterity issues and make the process easier. These include using electric toothbrushes or toothbrushes with easy-to-grip handles, as well as floss picks or water flossers for those with difficulty using traditional floss. Integrating health and social care and educating and empowering patients with the support of family or caregivers are also crucial. Additionally, building a workforce prepared for geriatric needs and ensuring affordable access through policy are crucial for long-term improvement.

## 5. Conclusions

In conclusion, MetS and periodontitis are prevalent among the elderly population. Our data provide direct evidence that MetS is a significant independent risk factor for periodontal disease in the elderly and underscore the need for periodontal care in older individuals.

## Figures and Tables

**Table 1:** Subject characteristics.

	MetS Group n = 32	Healthy Group n = 33	*p*-Value
Age Ave. (SD) years	78.12 (7.43)	73.18 (8.48)	0.01
Gender (male %)	53	45	0.53
Race			0.15
Asian %	15.63	12.12	
Black %	9.38	0	
Hispanic %	9.38	6.06	
White %	65.63	81.82	
BMI	34 (32–35)	26 (24–27.5)	<0.0001
Waist circumference	42 (39–44)	31 (30–33)	<0.0001
Diabetes %	100	0	
Hypertension %	100	0	
Current smoker	6.25	3.03	0.53

Data presented as mean (SD), percentage, or median (interquartile range). To test the differences between MetS and healthy controls, Student’s *t*-test was used for age and Wilcoxon two-sample test was used for all other continuous variables. Chi-square test was used for categorical variables.

**Table 2:** Periodontal measures.

	MetS Group n = 32	Healthy Group n = 33	*p*-Value
PI%	45.5 (23–66.25)	42 (23–65.5)	0.89
PD% (≥4 mm)	7.17 (1.86–18.76)	1.11 (0–4.64)	0.001
BOP%	21.5 (5–35.25)	4 (2–16.25)	0.0005
ABL average (mm)	3.24 (2.52–4.07)	2.53 (2.03–3.54)	0.03
ABL % ≥ 4 mm	26.38 (12.21–49.45)	12.24 (0–34.09)	0.02
Missing teeth (number)	5.5 (3–7)	5 (1–8.5)	0.55

Data presented as median (interquartile range). To test the differences between MetS and healthy controls, Wilcoxon two-sample test was used.

**Table 3:** Linear regression analyses testing the association of MetS with periodontal measures, while adjusting for demographics, cigarette smoking, and plaque score.

	Model 1 Demographics	Model 2 Cigarette Smoking	Model 3 Plaque Score
PD % ≥ 4 mm	0.31 *	0.39 **	0.40 **
BOP%	0.41 **	0.45 **	0.42 **
ABL average (mm)	0.29 *	0.24 *	0.28 *
ABL % ≥ 4 mm	0.31 *	0.26 *	0.29 *

Model 1 adjusts for demographics including age, sex/gender, and race/ethnicity. Model 2 adjusts for cigarette smoking. Model 3 adjusts for oral hygiene (plaque score). Data presented as standardized beta coefficients. * *p* < 0.05. ** *p* < 0.01.

## Data Availability

The data published in this study are available upon reasonable request from author A.K.

## Abbreviations

The following abbreviations are used in this manuscript:

MetS	Metabolic Syndrome
